# Undergraduate medical students’ perspectives on research education and their confidence in research skills: a cross-sectional study from Palestine

**DOI:** 10.1186/s12909-025-07586-w

**Published:** 2025-07-04

**Authors:** Ahmad Rjoub, Motaz Daraghma, Yazan Dumaidi, Mohammad A. Nour, Dalya Abusnaina, Tareq Fiqyat, Hamza A. Abdul-Hafez, Fadi Zaben, Malik Zaben

**Affiliations:** 1 Medical Education Department, International Medical Education Trust, 2000 - Palestine, Ramallah, Palestine; 2 St. Luke’s Health System, University of Missouri at Kansas City, Kansas City, MO USA; 3 Department of Medicine, Faculty of Medicine and Health Sciences, An- Najah National University, Nablus, Palestine; 4 Faculty of Medicine, Arab American University of Palestine, Jenin, Palestine; 5 Department of Medicine, College of Medicine and Health Sciences, Palestine Polytechnic University, Hebron, Palestine; 6 Department of Medicine, Faculty of Medicine, Al-Quds University, Jerusalem, Palestine; 7 Department of Nursing and Midwifery, Faculty of Medicine and Health Sciences, An-Najah National University, Nablus, Palestine

**Keywords:** Confidence, Education, Perspectives, Research skills, Undergraduate medical students

## Abstract

**Objective:**

Undergraduate medical research training is fundamental for developing future physicians’ research competencies and promoting evidence-based practice. This study aimed to evaluate Palestinian medical students’ perspectives on the adequacy of research education and examine their confidence in research skills in an attempt to improve research teaching.

**Methods:**

A cross-sectional, survey-based study design was used. An anonymous survey, consisting of two newly developed questionnaires that were reviewed by a panel of experts for content relevance and clarity, was used for data collection. After piloting, snowball sampling was used to survey students from 5 out of 7 medical schools in the country. Descriptive analysis and inferential statistics using chi-square tests, independent t-tests, one-way ANOVA, and Pearson’s correlation tests were used.

**Results:**

A total of 633 students participated in this study, representing 5 out of 7 medical schools in the country. The majority of students recognized curricular research training courses and the development of basic research skills as important milestones in their undergraduate education (85.3% and 83.4%, respectively). However, most students believed that they had received inadequate research training, with a mean Students’ Perspectives on the Adequacy of Research Education (SPARE) score of 5.53 (± 2.67) out of 11 (50.3%). Importantly, basics of research and research methodology (46.9%), study design (49.6%), methods of data collection (49.6%), data analysis (44.4%), and academic writing (43%) were reported by the students as key areas that are inadequately covered. Moreover, students reported a low level of confidence in their research skills, with a mean confidence score of 37.38 (± 10.02) out of 70 (53.4%). The SPARE scores were weakly correlated with the Students’ Confidence scores, *r* = 0.251, 95% CI (0.176 to 0.323).

**Conclusion:**

Palestinian medical students highly recognize the importance of research training. However, they feel that the current in-curriculum research training courses are inadequate to enhance their research skills. This study highlighted the pressing need for Palestinian medical schools and others with similar curricula to consider a more efficient curriculum development and delivery to meet students’ needs and improve confidence in their own research skills as future clinicians.

**Clinical trial number:**

Not applicable.

**Supplementary Information:**

The online version contains supplementary material available at 10.1186/s12909-025-07586-w.

## Background

With the current emphasis on evidence-based clinical practice, research training as an integral part of undergraduate medical education has been increasingly recognized as an instrumental mean in preparing future physicians to critically appraise scientific literature, conduct impactful studies, and integrate research into their clinical practice [1, 2, 3]. However, the shortage of qualified physician-scientists has significantly hindered fulfilling this objective [4]. In this context, multiple studies have suggested that integrating research training into undergraduate medical curricula is paramount, as it helps learners develop a sense of scientific inquiry, enhances students’ analytical abilities, and encourages lifelong learning at an early stage [1, 2, 3, 5, 6, 7]. In addition, the World Federation for Medical Education (WFME) has clearly stated that the principles of scientific method and medical research in the curriculum need to be incorporated into the basic medical education curriculum [8]. Similarly, scientific research as an important part of scholarship and professional careers has been recognized by the Association of Medical Education in Europe (AMEE) in their guide on developing research skills for medical students [9].

Despite that, the exact timing, extent of subject coverage, and means of introducing research into medical curricula remain debatable. Early exposure to and involvement of medical students in research has been reported to influence the future physicians’ scientific activity in the long term [10, 11, 12, 13, 14]. This is particularly important to ensure that future clinical investigators are competent, because low-quality research is not just a waste of researchers’ time and institutions’ resources, but can negatively impact clinical outcomes and public health [15]. In response to that, we have witnessed increasingly early integration of research training in undergraduate medical schools across the globe. No doubt, the success of such a critical step needs to have in place enough data to understand students’ perspectives on research and largely depends on the available expertise and resources. This could be particularly challenging for medical schools in low- and middle-income countries (LMIC), including Palestine. Like many of LMIC, Palestinian medical schools integrate research training on multiple levels, including courses such as epidemiology, biostatistics, and research methods, and a graduation thesis as mandatory requirements for obtaining a medical degree. However, the effectiveness and impact of the current research training programs in the medical school curriculum in Palestine remain unclear, taking into consideration political instability, restricted resources, and fragmented infrastructure [16].

Literature on the Palestinian medical schools’ efforts to develop students’ research skills, the students’ perspectives on the adequacy of the research training they receive, and the students’ confidence in applying research skills is limited. This study aims to evaluate Palestinian medical students’ perspectives on the current state of research training in their undergraduate programs and examine their self-reported confidence in essential research skills to identify potential areas for improvement in research training at Palestinian medical schools. By understanding students’ experiences and confidence in their research skills, findings from this study informs curriculum reforms in Palestine and thereby places future physicians in a better position to address healthcare system challenges through enhanced research competencies and evidence-based practice.

## Methods

### Setting and study design

A cross-sectional survey-based study design was used to explore clinical-phase medical students’ perspectives on research training in undergraduate medical education and their self-reported confidence in applying basic research skills. Medical students in Palestinian medical schools receive curricular research training starting from the third year. The study population consisted of clinical-phase medical students from all the Palestinian medical schools in the West Bank who completed the curricular research training. Due to logistical constraints and the disruption of medical education in the Gaza Strip due to the ongoing conflict during the time of the study, medical schools in the Gaza Strip were excluded.

### Study sample and participants recruitment

A total of 633 medical students were recruited for this study using a convenient, snowball sampling technique. The use of snowball sampling was due to logistical limitations and the absence of a centralized student database. Initial participants disseminated the survey among their peers, facilitating a broad reach across geographically dispersed medical schools in the West Bank. This method facilitated the efficient recruitment of a large and diverse sample, optimizing institutional representation despite restricted direct access to students. Medical students in their third academic year or higher, who have completed their curricular research training, were included in this study. Students who did not complete the curricular research training were excluded from the study. The Raosoft^®^ Sample Size Calculator was used to determine the minimum sample size, using the following formula: n = N * x / ((N-1) E2 + x), where N represents the total population size, and x is derived from the Z-score of 1.96 (for a 95% confidence level). Given an estimated population size (N) of 5,000, a response distribution of 50%, and a margin of error (E) of 5%, the minimum recommended sample size was calculated to be 357. The potential bias from the use of a non-probability sampling technique was minimized by recruiting an adequate sample representing medical students from most of the medical schools in the country, therefore, enhancing the generalizability and reliability of the findings.

### 2.3. Data collection tool

The data were collected by the research team through an online anonymous survey, built using Google Forms, and the responses were transferred to Google Spreadsheets. The duration for data collection was from February 2025 to March 2025. The survey had a total of 31 questions and consisted of three sections: sociodemographic characteristics and two newly designed questionnaires. The first section included 6 questions regarding the sociodemographic variables and students’ previous research experience. The second section was a questionnaire that contained 11 questions on the students’ perspectives on the adequacy of research education in their undergraduate medical education. This questionnaire used 11 questions with (yes/no) answers instead of statements with agree/disagree items to engage the participants with the questionnaire. The third section was a questionnaire that contained 14 questions on the students’ self-reported confidence levels in applying basic research skills. The self-reported confidence score questionnaire consisted of 14 questions with Likert-type options (from not at all confident to extremely confident) to allow the participants to self-report their confidence in applying research skills. A panel of two expert researchers and medical educationists independently reviewed the questionnaires to ensure their clarity and content relevance. The questionnaires were written in English, the official teaching language in Palestinian medical faculties. A copy of the survey is attached (Supplementary Material 1). Before distributing the form, a pilot study was conducted, enrolling 40 participants from the targeted population. The participants’ understanding of the items was assessed, and the necessary changes were made to enhance the clarity of the items. The estimated time to complete the questionnaire was 4–5 min. The internal consistency of the questionnaire items was assessed using the 40 responses, with Cronbach’s alpha of 0.816 for the Perspectives score and 0.969 for the confidence score, corresponding to a very good and excellent internal consistency, respectively. The responses from the pilot study were not included in the analysis of the main study.

### Study variables

The sociodemographic variables of the study included gender, year of study, and university. Variables regarding the students’ experience in research were also included as potential confounders. The outcome variables were two scores calculated from the two questionnaires: [1] the Students’ Perspectives on the Adequacy of Research Education (SPARE) Score (0–11), calculated by the 11 dichotomous Yes/No items (1 = Yes, 0 = No), with scores ≥ 8/11 (≥ 70%) indicating a perception of adequate research education inclusion in the medical curricula, and [2] the Students’ Confidence in Basic Research Skills (SCBRS) Score (14–70), calculated from the 14 Likert-scale items (1 = Not at all confident, 2 = Slightly confident, 3 = Moderately confident, 4 = Quite confident, and 5 = Extremely confident), where scores ≥ 56/70 (≥ 80%) reflected an adequate confidence. While no exact cutoffs for the Students’ Perspectives and Confidence Scores were found in the literature, we selected 70% as a limit for the Students’ Perspectives on the Adequacy of Research Education Score based on educational survey standards (60–80% for favorable perspectives), and 80% as a threshold for confidence scores, emphasizing more strict competency measures for basic research skills for medical students.

### Data analysis

The Statistical Package for Social Sciences (SPSS) version 27.0 was used for data analysis. Descriptive statistics were applied to the sociodemographic variables, research experience variables, perspective scores, and confidence scores, which were summarized as frequencies, percentages, means, and standard deviations. Inferential statistics were applied to assess possible associations between the variables. Chi-square tests were conducted to assess associations between categorical variables, including the sociodemographic variables, research experience variables, and items of the research education adequacy perspectives questionnaire [17]. Independent sample t-tests to compare mean scores between predefined groups (male vs. female, working on a research project vs. not, and publishing a research article vs. not) [18]. One-way ANOVA tests were conducted to compare the mean scores between the groups of students from the fourth, fifth, and sixth years [18]. A Pearson correlation test was conducted to evaluate the relationship between perspectives and confidence scores [19]. The significance level was set to 5% ( α = 0.05) for all statistical tests.

### Ethical consideration

The study was conducted in accordance with the Declaration of Helsinki. An ethical approval to conduct this study was obtained from the Institutional Review Board (IRB) at the Arab American University of Palestine (AAUP), Ramallah Campus (IRB code: R-2025/A/14/N). The opening section of the online survey contained the study information sheet and an online informed consent. The participants were informed that their participation in the study was voluntary and that they could withdraw from the study at any time. No personal information was collected from the participants.

## Results

### Sociodemographic characteristics of the participants

Totally, 633 medical students participated in this study, representing 5 out of 7 medical schools in the country, with a nearly even distribution. Nearly 52.8% of the students were in the fourth year, while 25.8% and 21.5% of the students were in their fifth and sixth years, respectively. All of the participants have received formal research training as part of the curriculum. Moreover, while about 59% of the students participating in this study reported that they had worked on a research project, only 25.8% of them reported that they had published a research article before (Table 1).

**Table 1:** Sociodemographic characteristics of medical students participating in the study (*N* = 633)

Variable	Frequency (*N*)	Percentage (%)
**Gender**		
Male	337	53.2
Female	296	46.8
**Year of Study**:		
Fourth year	334	52.8
Fifth year	163	25.8
Sixth year	136	21.5
**University**:		
Hebron University	158	25
Palestine Polytechnic University	153	24.2
Arab American University	132	20.9
An-Najah National University	110	17.4
Al-Quds University	80	12.6
**Have you received any formal research education (Research Methodology Course) in medical school?**		
Yes	633	100
**Have you worked on a research project before?**		
Yes	375	59.2
**Have you published any research articles before?**		
Yes	163	25.8
**Total**	633	100

### Perspectives of students on the adequacy of research education in their curriculum

The total students’ perspectives on the adequacy of research education (SPARE) score was 5.53 (± 2.67) out of 11, representing only 50.3% of the maximum score. Fourth-year students had significantly higher perspectives scores than fifth and sixth-year students (5.91 vs. 5.05 vs. 5.2, p-value < 0.001). Other variables, such as gender, working on a research project, and publishing a research article, did not affect students’ perspectives on the adequacy of research education (Table 2). About 85.3% of the students have stated that research education is important in undergraduate medical education, and 83.4% of them believed that medical students should develop a set of basic skills in medical research. Approximately 62% of the students believe that the amount of research training and education in their medical school is inadequate. Importantly, the majority of students who participated in the study believed that their medical curricula inadequately cover the basics of research and research methodology, types of study designs, methods of data collection, data analysis, results presentation and visualization, and academic writing. Moreover, most of the students reported that they believe their medical curricula include an inadequate level of practical training in research, mentorship, and support. Multiple differences in the perspectives on the various categories of the score were found between the groups (Table 3).

**Table 2:** Medical students’ perspectives on the adequacy of research education (SPARE) score in the undergraduate curriculum (*N* = 633)

**Variable**	**Perspectives Score**			
	**Mean**	**SD**	% **of the maximum score**	*P*-value ^α^
**Gender**:				
Male	5.46	± 2.38	49.6%	0.463
Female	5.61	± 2.96	51.0%	
**Worked on a research project**:				
Yes	5.47	± 2.41	49.7%	0.48
No	5.63	± 3	51.2%	
**Published a research article**:				
Yes	5.57	± 2.31	50.6%	0.824
No	5.52	± 2.78	50.2%	
**Variable**	**Perspectives Score**			
	**Mean**	**SD**	**% of the maximum score**	*P-value* ^β^
**Year of Study**:				
Fourth	5.91	± 2.63	53.7%	< 0.001*
Fifth	5.05	± 2.75	45.9%	
Sixth	5.2	± 2.54	47.3%	
**Total Students’ Perspectives on the Adequacy of Research Education Score**	**5.53**	**± 2.67**	**50.3%**	-

*Statistically significant (< 0.05)

^α^ Independent Samples T-test

^β^ One-way ANOVA test

**Table 3:** Medical students’ perspectives on the adequacy of research education in the undergraduate curriculum, answers to the questionnaire items (*N* = 633)

Variable	*n* (%)	Gender			Year of Study				Worked on a Research Project			Published a Research Article		
Item		Male	Female	*P*-value ^α^	Fourth	Fifth	Sixth	*P*-value ^α^	Yes	No	*P*-value ^α^	Yes	No	*P*-value ^α^
Research education is important in undergraduate medical education. (Yes)	540 (85.3%)	253 (75.1%)	287 (97%)	< 0.001*	261 (78.1%)	156 (95.7%)	123 (90.4%)	< 0.001*	295 (78.7%)	245 (95%)	< 0.001*	120 (73.6%)	420 (89.4%)	< 0.001*
Medical students should develop a set of basic skills in medical research. (Yes)	528 (83.4%)	240 (71.2%)	288 (97.3%)	< 0.001*	256 (76.6%)	157 (96.3%)	115 (84.6%)	< 0.001*	280 (74.7%)	248 (96.1%)	< 0.001*	100 (61.3%)	428 (91.1%)	< 0.001*
The amount of research training and education in your medical school is adequate. (No)	393 (62.1%)	192 (57%)	201 (67.9%)	0.005*	179 (53.6%)	126 (77.3%)	88 (64.7%)	< 0.001*	215 (57.3%)	178 (69%)	0.003*	87 (53.4%)	306 (65.1%)	0.008*
Your medical curriculum adequately covers the basics of research and research methodologies. (Yes)	297 (46.9%)	157 (46.6%)	140 (47.3%)	0.858	164 (49.1%)	73 (44.8%)	60 (44.1%)	0.505	175 (46.7%)	122 (47.3%)	0.878	85 (52.1%)	212 (45.1%)	0.121
Your medical curriculum adequately covers the different types of research study designs. (Yes)	314 (49.6%)	166 (49.3%)	148 (50%)	0.852	174 (52.1%)	77 (47.2%)	63 (46.3%)	0.411	196 (52.3%)	118 (45.7%)	0.106	93 (57.1%)	221 (47%)	0.027*
Your medical curriculum adequately covers the different methods of data collection. (Yes)	315 (49.6%)	172 (51%)	142 (48%)	0.441	180 (53.9%)	75 (46%)	59 (43.3%)	0.067	187 (49.9%)	127 (49.2%)	0.874	78 (47.9%)	236 (50.2%)	0.604
Your medical curriculum adequately covers biostatistics and data analysis. (Yes)	281 (44.4%)	154 (45.7%)	127 (42.9%)	0.481	172 (51.5%)	58 (35.6%)	51 (37.5%)	< 0.001*	155 (41.3%)	126 (48.8%)	0.062	74 (45.4%)	207 (44%)	0.764
Your medical curriculum adequately covers the different methods of results visualization. (Yes)	233 (36.8%)	139 (41.2%)	94 (31.8%)	0.014*	152 (45.5%)	40 (24.5%)	41 (30.1%)	< 0.001*	149 (39.7%)	84 (32.6%)	0.066	71 (43.6%)	162 (34.5%)	0.038*
Your medical curriculum adequately covers the basics of academic writing. (Yes)	272 (43%)	139 (41.2%)	133 (44.9%)	0.35	166 (49.7%)	59 (36.2%)	47 (34.6%)	0.001*	159 (42.4%)	113 (43.8%)	0.727	71 (43.6%)	201 (42.8%)	0.86
The research education in your faculty includes an adequate level of mentorship and support from the mentors or supervisors. (Yes)	267 (42.2%)	143 (42.4%)	124 (41.9%)	0.891	166 (49.7%)	54 (33.1%)	48 (34.6%)	< 0.001*	156 (41.6%)	111 (43%)	0.722	74 (45.4%)	193 (41.1%)	0.334
The research education in your faculty includes an adequate level of practical training. (Yes)	217 (34.3%)	132 (39.2%)	85 (28.7%)	0.006*	127 (38%)	37 (22.7%)	53 (39%)	0.001*	139 (37.1%)	78 (30.2%)	0.075	66 (40.5%)	151 (32.1%)	0.053

*Statistically significant (< 0.05)

^α^ Chi-square test

### Students’ confidence in applying basic research skills

**Fig. 1:**
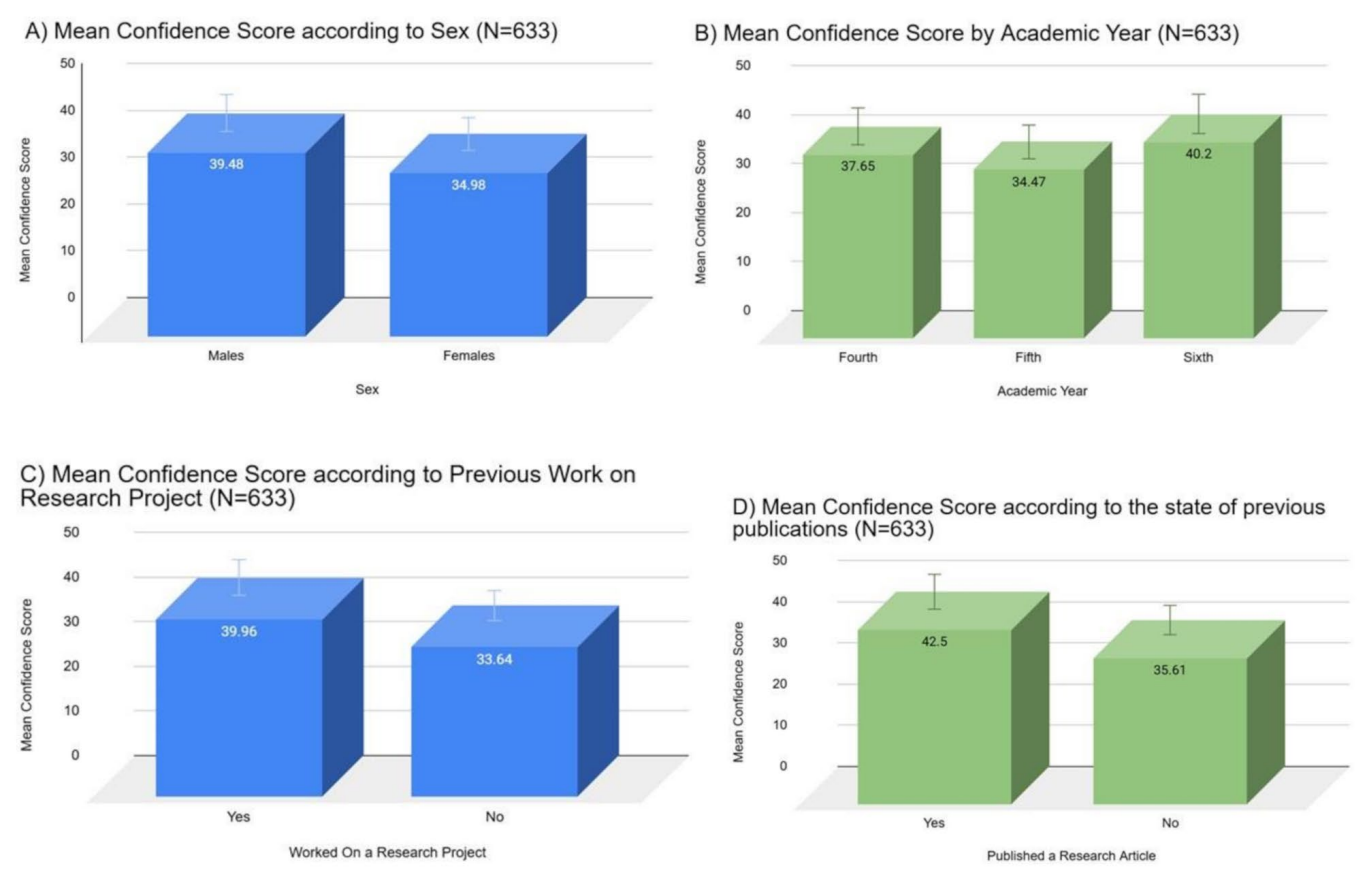
Mean confidence scores according to sex **(A)**, academic year **(B)**, previous work on a research project **(C)**, and previous research publication **(D)**

The overall students’ confidence in applying basic research skills was inadequate, with a mean of the total Students’ Confidence score of 37.38 (± 10.02) out of 70, representing 53.4% of the maximum score. Males had slightly higher confidence scores than females (39.48 vs. 34.98, p-value < 0.001). Fourth-year students had lower confidence scores than sixth-year students, but higher than fifth-year students (37.65 vs. 34.47 vs. 40.2, p-value < 0.001). Students who worked on a research project and those who published a research article had higher confidence scores (39.96 vs. 33.64, and 42.50 vs. 35.61, respectively, p-value < 0.001) (Fig. 1). Moreover, the mean scores in the different categories of the total confidence score were different between genders, the study year, experience in working on a research project, and publishing a research article. Students displayed an inadequate confidence level in all of the basic research skills categories, with significantly low confidence in using software for analysis and reference management (2.49 ± 1.20 out of 5), critical appraisal (2.50 ± 1.08 out of 5), data analysis (2.52 ± 1.11 out of 5), and scientific writing (2.55 ± 1.06 out of 5) (Table 4).

**Table 4:** Comparison of students’ Self-Reported confidence in applying basic research skills across gender, year of study, and research experience (*N* = 633)

Variable	Mean (± SD)	Gender			Year of Study				Worked on a Research Project			Published a Research Article		
Item		Male	Female	*P*-value ^α^	Fourth	Fifth	Sixth	*P*-value ^β^	Yes	No	*P*-value ^α^	Yes	No	*P*-value ^α^
Conducting a research project on their own.	2.57 (± 1.06)	2.71	2.4	< 0.001*	2.54	2.4	2.85	< 0.001*	2.8	2.23	< 0.001*	2.96	2.44	< 0.001*
Formulating a good research question.	2.79 (± 1.01)	2.89	2.68	0.009*	2.72	2.75	3.04	0.008*	2.98	2.53	< 0.001*	3.03	2.71	0.002*
Conducting a literature search and review.	2.79 (± 1.09)	2.91	2.65	0.002*	2.69	2.78	3.03	0.011*	3.02	2.45	< 0.001*	3.21	2.64	< 0.001*
Choosing the appropriate study design and research methodology.	2.73 (± 1.09)	2.93	2.5	< 0.001*	2.73	2.53	2.96	0.003*	2.95	2.41	< 0.001*	3.29	2.54	< 0.001*
Writing a research proposal.	2.78 (± 1.09)	2.91	2.64	0.002*	2.73	2.69	3.03	0.012*	3.01	2.45	< 0.001*	3.1	2.67	< 0.001*
Determining the target population and choosing the appropriate sampling technique.	2.80 (± 1.09)	2.87	2.71	0.063	2.81	2.69	2.92	0.185	2.93	2.61	< 0.001*	3.1	2.69	< 0.001*
Choosing the appropriate data collection method.	2.75 (± 1.10)	2.88	2.6	0.002*	2.72	2.63	2.95	0.035*	2.89	2.55	< 0.001*	3.02	2.66	< 0.001*
Conducting data analysis.	2.52 (± 1.11)	2.7	2.31	< 0.001*	2.64	2.51	2.65	< 0.001*	2.67	2.3	< 0.001*	2.85	2.41	< 0.001*
Ability to present and visualize the results.	2.64 (± 1.07)	2.77	2.5	0.001*	2.68	2.43	2.82	0.005*	2.77	2.47	< 0.001*	2.94	2.54	< 0.001*
Interpreting the results of their study.	2.82 (± 1.08)	2.95	2.67	0.001*	2.86	2.57	3.03	0.001*	2.99	2.57	< 0.001*	3.08	2.73	< 0.001*
Writing and submitting a research manuscript for publication.	2.55 (± 1.06)	2.74	2.34	< 0.001*	2.62	2.21	2.8	< 0.001*	2.76	2.26	< 0.001*	2.89	2.36	< 0.001*
Critically appraising published research articles.	2.50 (± 1.08)	2.71	2.26	< 0.001*	2.59	2.18	2.67	< 0.001*	2.68	2.23	< 0.001*	3.1	2.37	< 0.001*
Presenting research findings at conferences.	2.60 (± 1.09)	2.7	2.48	0.012*	2.66	2.34	2.78	< 0.001*	2.72	2.43	< 0.001*	2.91	2.5	< 0.001*
Using softwares like SPSS, EndNote, or Mendeley Reference Manager.	2.49 (± 1.20)	2.76	2.19	< 0.001*	2.63	2.08	2.66	< 0.001*	2.75	2.14	< 0.001*	3	2.32	< 0.001*
**Total Confidence Score**	**37.38 (± 10.02)**	39.48	34.98	< 0.001*	37.65	34.47	40.2	< 0.001*	39.96	33.64	< 0.001*	42.5	35.61	< 0.001*

*Statistically significant (< 0.05)

^α^ Independent Samples T-test

^β^ One-way ANOVA test

### Correlation between students’ perspectives score and confidence score

**Fig. 2:**
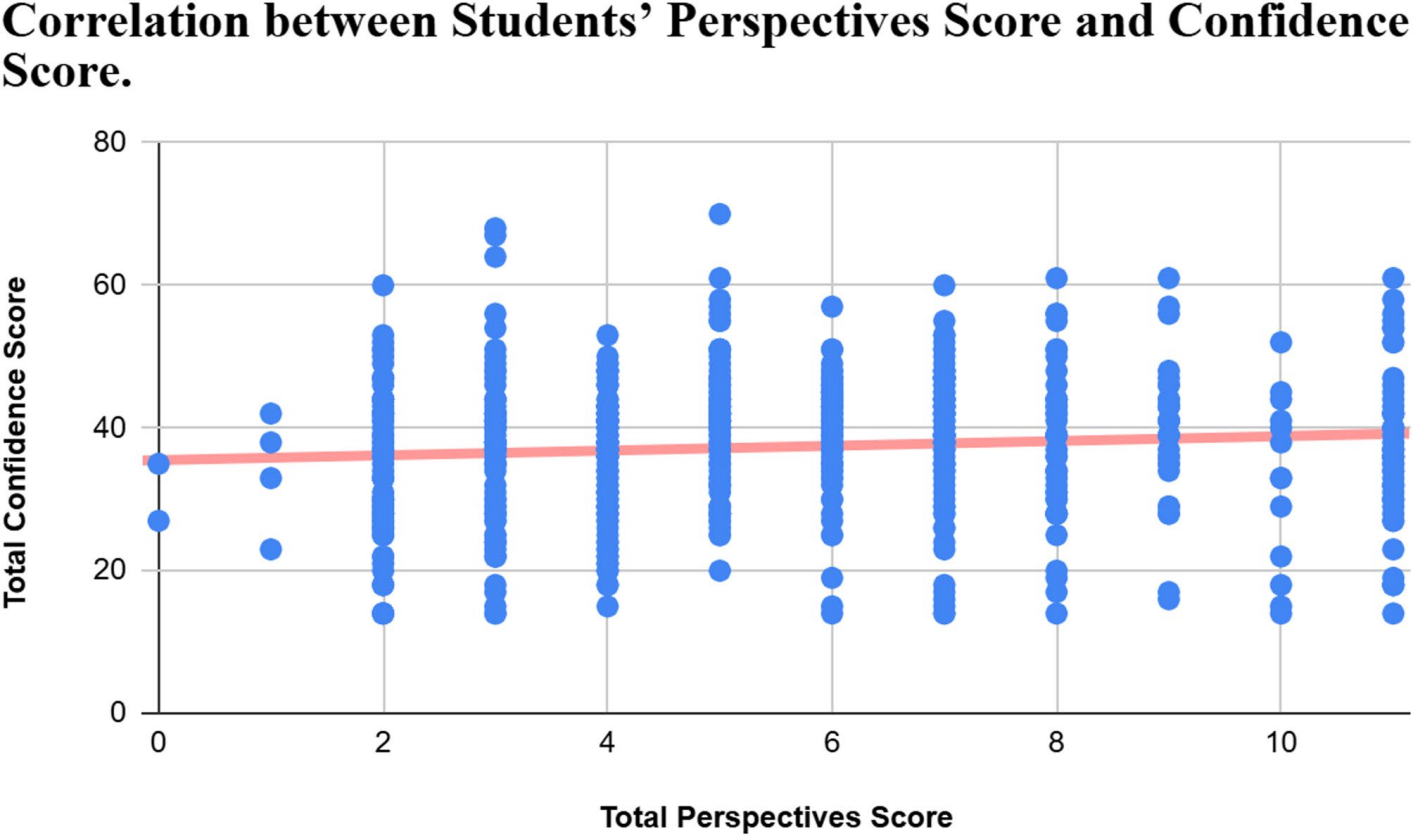
Scatter plot for the correlation between Students’ Perspectives and Confidence scores

Although weak, a significant positive correlation was found between the Students’ Perspectives on the Adequacy of Research Education score and Students’ Confidence in Basic Research Skills score, Pearson’s correlation coefficient = 0.251, 95% CI (0.176 to 0.323), p-value < 0.001 (Table 5; Fig. 2).

**Table 5:** Correlation between students’ perspectives score and confidence score

Variables	Students’ Perspectives on the Adequacy of Research Education score		
**Confidence Score**	***r***	**P-value** α	**95% CI**
	0.251	< 0.001*	(0.176–0.323)

*Statistically significant (< 0.05)

α Pearson correlation test

## Discussion

This study is the first of its type to provide in-depth insights into the Palestinian medical students’ perspectives on the adequacy of research training in their curriculum. Additionally, our study assessed students’ self-reported confidence levels in applying basic research skills in an attempt to inform the literature on how to improve research training at the undergraduate stage in a low-to-middle-income country where traditional 6-year medical curricula are widely used. We report for the first time that the majority of Palestinian students recognize the importance of research training for them as future doctors. However, students surveyed felt that the current in-curricular training is inadequate, and as a result, they have very low confidence in their own research skills. This study highlights the pressing need for further research looking into means and venues to improve research training in currently implemented curricula. The results of this study highlighted critical gaps in the current curricular research training programs in undergraduate medical schools in Palestine, based on the students’ perspectives and confidence. Our findings indeed inform curriculum developers in Palestinian medical schools and others in the region and across the world regarding the research education strategies that should be adopted in the curriculum.

### Students’ perspectives on the adequacy of the research education in their curriculum

The mean students’ perspectives on the adequacy of research education score was only 50.3% of the maximum score. This is below the threshold for perception of adequate integration of research education (70% or more). The findings of this study revealed that while most medical students acknowledge the importance of integrating research education into undergraduate medical education and the importance of developing a set of basic skills in medical research, they perceive that the amount of research education integrated into their curricula was inadequate. This finding aligns with the results of the global survey conducted by *Pierre et al.* [20].

The mean scores of students’ perspectives on the adequacy of research education did not significantly differ between both genders, students who worked on a research project versus students who did not work, and students who published a research article versus students who did not publish. This finding highlights that these variables did not interfere with the students’ perception of inadequate research education in their curricula. Interestingly, a consensus was found among the students on the inadequacy of the curricular research training, regardless of their past research activity, which may indicate that the students’ research activity is not related to the current status of the curricular research training, and students may seek other extracurricular sources for research training and enhancing their research records. Additionally, students’ exposure to research activities in the form of practical research projects or publications may lead to identifying gaps in the curricular training they received. The impact of extracurricular research training on the students’ perception of curricular research training and their research skills remains unclear. On the other hand, fourth-year students had a slightly higher perspective score than fifth and sixth-year students. This can be attributed to the optimism linked to earlier educational stages before undertaking advanced research tasks that reveal gaps in their preparedness to engage in actual research work.

The study findings highlight significant gaps in the adequate coverage of essential research competencies in the medical student curricula from the students’ point of view. These essential skills include the basics of research, research methodology, types of study designs, methods of data collection, data analysis, results presentation and visualization, and academic writing. This was in line with a study that assessed the importance of research competencies in the German medical curricula, in which students felt that improvements in the coverage and teaching of statistics and academic writing are needed and expressed a desire for more comprehensive research training [21]. Filling the gaps in the curriculum and improving the coverage of these essential research competencies are crucial to promoting the students’ involvement in scientific research by providing sufficient training [22].

Moreover, Palestinian medical students believe that the research training in their schools lacks adequate support, mentorship, and practical training. The lack of sufficient supervision was reported in a previous study as a significant barrier to medical students’ involvement in research [23]. Additionally, the presence of research mentors was identified as a promoter of Palestinian resident doctors’ engagement in research [24]. The role of effective mentorship in research in supporting the personal and professional development of medical students is crucial, especially in competitive residency programs [25]. While the consensus is that mentees should be more proactive in developing and maintaining the mentoring relationship, successful mentoring in academic medicine relies on mentors’ and mentees’ commitment and interpersonal skills, in addition to a facilitating environment created and maintained by their institutions [26, 27]. Targeted mentor training programs are vital to prepare the mentors to provide effective guidance and personalized support [28, 29]. *Pfund et al.* in their randomized trial concluded that a competency-based mentor training program can effectively develop the mentorship skills of faculty mentors of trainees involved in research [30]. The students’ perceived need for adequate practical training in research was in line with the findings of a previous study from Germany [31]. Notably, more practical and competency-based research training was shown to be an effective tool in teaching research methodology for undergraduate medical students, highly satisfactory for the students [32, 33]. Therefore, addressing the perceived inadequacies in mentorship and practical training within Palestinian medical schools is important for improving the research education in these schools and developing research competencies and professional careers of future physician-scientists, ultimately contributing to improving the quality of the healthcare system in Palestine.

### Students’ confidence in applying basic research skills

The findings of this study showed that medical students demonstrated particularly low confidence in applying basic research skills. Similar results of an unsatisfactory level of confidence in research skills were reported in a previous study from Germany [31]. Male students scored higher on self-rated confidence scores than female students, aligning with the results of previous studies, where male students appeared to be more confident in their research skills [31, 34]. Similar findings were also reported in medical education literature, indicating that female medical students tend to report lower self-confidence scores in their clinical skills, possibly due to their anxiety when it comes to their competencies [35]. In addition, students with experience in working on research projects or published research articles showed higher confidence scores, suggesting a potential benefit of research opportunities and practical research experiences playing an important role in medical students’ research skills development. This is also aligned with previous research findings that emphasized the importance of having practical exposure in building students’ research skills and confidence [36]. However, despite the differences between the groups, the overall confidence in applying research skills was inadequate, highlighting the need for improving research education in undergraduate medical schools in Palestine and adopting a more practical, competency-based approach to build stronger competencies and elevate future physicians’ confidence.

Students displayed a significantly low confidence level in all of the basic skills included in the questionnaire, particularly skills requiring a higher level of critical and analytical thinking, such as data analysis, critical appraisal, scientific writing, and using software for analysis and reference management. This finding aligns with previous studies [31, 34]. Highlighting these competencies as targets for potential improvement in the curricular research training in Palestine, we expect that adequate and effective training on the abovementioned competencies would enhance the overall students’ confidence in their research skills. A systematic review by *Albarqouni et al.* concluded that the quality of medical and public health research publications from Palestinian institutions has improved over the past decade; however, it remains below the accepted quality when judged against the international reporting guidelines [37]. Moreover, several studies reported that training programs focused on critical appraisal and scientific writing significantly improved medical students’ confidence and performance in these essential competencies [38, 39]. Overall, there is a pressing need for a targeted improvement of the research training curricula and the adoption of a more practical, competency-based approach, based on the needs of Palestinian medical students and practicing physicians, to improve the quality of research produced in Palestinian institutions.

### Recommendations

Based on our findings, we suggest the following recommendations to support Palestinian and other medical schools using the same traditional curriculum in addressing their students’ needs in terms of research competencies. This could help enhance their abilities to equip their students with the essential research skills, to meaningfully contribute to the advancement of medical sciences, and to improve the Palestinian healthcare system through an evidence-based approach. First, our study findings highlighted the need for a more structured, standardized, and comprehensive integration of research training in undergraduate medical schools. Henceforth, we recommend that the medical schools using this 6-year curriculum conduct a curriculum reform to address the students’ needs and ensure adequate and in-depth coverage of essential skills, including research methodology, study design, data analysis, academic writing, and critical appraisal. Secondly, using a competency-based approach for research training is recommended and can be useful in developing the research competencies of medical students and future physicians. Additionally, using the AMEE guide on developing research skills in medical students can be helpful for Palestinian medical schools, especially for curriculum development, reform, integrating essential skills, and assessment methods [9]. Thirdly, a pressing need for enhancing the research mentorship and the practical part of the research training in medical schools was identified. Therefore, addressing the students’ perceived lack of adequate guidance, mentorship, and practical research training is crucial to develop their research competencies. This can be done by integrating a competency-based approach for research training, establishing and maintaining a supportive environment of research mentorship by medical schools, and designing and providing targeted competency-based mentor training programs. Finally, continuous monitoring and evaluation of the research training’s impact on students’ experience and confidence in their research competencies, using standardized feedback surveys, and tracking the students’ scientific performance and publication rates, is vital to guide future educational interventions.

### Future directions

Future research should be directed toward tracking the impact of educational interventions in research training on the students’ scientific productivity and future careers. This could help provide insights into whether improving research training in Palestinian medical schools can have a positive impact on the scientific advancement of graduates, increasing their meaningful publications, and contributing to improving the healthcare system. Moreover, studies can focus on the comparison between different research training programs and curricula to evaluate the effectiveness of these programs across Palestinian medical schools. Moreover, the impact of effective mentorship programs on the students’ competencies, productivity, and performance can also be a target for future studies. Additionally, future research can focus on identifying the potential reasons for the gender differences in confidence in research skills and aim for interventions to close this gender gap. Finally, additional research is needed to assess the potential role that extracurricular research training could play in complementing formal curricular research training.

### Strengths and limitations

This study has some strengths, including the use of newly developed questionnaires, the large and adequate sample size, and the inclusion of all the medical schools in the West Bank of Palestine. However, despite the significance of the study findings, several limitations should be noted. First, the data collection method used was mostly subjective, relying on the students’ perspectives and self-reported confidence, leading to a potential reporting bias and social desirability bias, as students could overestimate their perspectives on the adequacy of the research training and their confidence. Using more objective assessment methods could enhance the findings of this study. Moreover, the use of a convenient sampling technique and the unfortunate exclusion of medical schools in the Gaza Strip due to logistical constraints may have led to a selection bias, limiting the generalizability of the study findings, however, we expect that the results might not differ significantly if medical schools from the Gaza strip were included, considering the relatively similar structure of research training among medical schools in Palestine. Finally, although the snowball sampling method might have affected participant characteristics, it allowed us to overcome institutional and logistical constraints by allowing us to collect a larger and more representative sample from most of the medical schools in Palestine. Future research might explore more representative sample techniques should institutional access or centralized registries become accessible.

## Conclusions

In conclusion, although research training has been integrated into all the Palestinian medical schools, its impact on the students’ perspectives and confidence in their research competencies has not been studied before. This study highlights the need for improvements in the research training provided to undergraduate medical students in Palestine, identifying the gaps and areas for potential improvement. While most of the students recognize the importance of research training, they feel that the amount of research training they receive is inadequate. Moreover, students report a lack of confidence in their research competencies. Future educational interventions by medical schools should focus on enhancing the integration of research training in their curricula, in-depth and more practical coverage of essential topics such as research methodology, study design, data analysis, academic writing, and critical appraisal, and shifting toward a competency-based approach in training. A pressing need for enhancing the mentorship in research was identified, which can be addressed by establishing and maintaining a supportive environment of research mentorship by medical schools and conducting targeted competency-based mentor training programs. Future research can focus on the long-term evaluation of the effectiveness of these educational interventions, comparisons between different research training programs, and closing the gender gap in confidence in research competencies. By addressing the students’ needs, medical schools can contribute to developing more solid research skills among medical students, producing more competent and well-trained physician-scientists, leading to enhancing the quality of clinical research products of Palestinian institutions, and ultimately, the quality of the healthcare system.

## Electronic supplementary material

Below is the link to the electronic supplementary material.

Supplementary Material 1

## Data Availability

The datasets used and/or analyzed during the current study are available from the corresponding author on reasonable request.

## Abbreviations

AMEE: Association for Medical Education in Europe International Association for Health Professions Education
LMIC: Low- and Middle-Income Countries
SCBRS: Students’ Confidence in Basic Research Skills
SPARE: Students’ Perspectives on the Adequacy of Research Education
WFME: World Federation for Medical Education
