# Supplementary material for: Undergraduate medical students’ perspectives on research education and their confidence in research skills: a cross-sectional study from Palestine
Source: BMC Med Educ. 2025 Jul 4;25:1005. doi: 10.1186/s12909-025-07586-w (PMC12228341; doi:10.1186/s12909-025-07586-w)
Supplement: Supplementary file 1 — Supplementary Material 1 [file 12909_2025_7586_MOESM1_ESM.docx]

**Supplementary Table**

**Copy of the Original Survey used in this Study**

| **Section 1: Sociodemographic Characteristics** | |
| --- | --- |
| **1** | **Gender:**   - Male - Female |
| **2** | **Year of Study:**   - Fourth-year - Fifth year - Sixth year |
| **3** | **University:**   - An-Najah National University - Al-Quds University (Main Campus in Abu Dis) - Al-Azhar University-Gaza - Islamic University of Gaza - Hebron University - Palestine Polytechnic University - Arab American University |
| **4** | **Residence type:**   - City - Village - Camp |
| **5** | **Have you received any formal research education (Research Methodology Course) in medical school?**   - Yes - No |
| **6** | **Have you worked on a research project before?**   - Yes - No |
| **7** | **Have you published any research articles before?**   - Yes - No |
| **Section 2: Perception of Research Education** | |
| **8** | **Do you believe that research education is important in undergraduate medical education?**   - Yes - No |
| **9** | **Do you believe that medical students should develop a set of basic skills in medical research?**   - Yes - No |
| **10** | **Do you believe that the amount of research training and education in your medical school is adequate?**   - Yes - No |
| **11** | **Do you believe your medical curriculum adequately covers the basics of research and research methodologies?**   - Yes - No |
| **12** | **Do you believe your medical curriculum adequately covers the different types of research study designs?**   - Yes - No |
| **13** | **Do you believe your medical curriculum adequately covers the different methods of data collection?**   - Yes - No |
| **14** | **Do you believe your medical curriculum adequately covers biostatistics and data analysis?**   - Yes - No |
| **15** | **Do you believe your medical curriculum adequately covers the different methods of results visualization?**   - Yes - No |
| **16** | **Do you believe your medical curriculum adequately covers the basics of academic writing?**   - Yes - No |
| **17** | **Do you believe that the research education in your faculty includes an adequate level of mentorship and support from the mentors or supervisors?**   - Yes - No |
| **18** | **Do you believe that the research education in your faculty includes an adequate level of practical training?**   - Yes - No |
| **Section 3: Confidence in Research Skills** | |
| **19** | **How confident are you in conducting a research project on your own?**   - Not at all confident - Slightly confident - Moderately confident - Quite confident - Extremely confident |
| **20** | **How confident are you in formulating a good research question?**   - Not at all confident - Slightly confident - Moderately confident - Quite confident - Extremely confident |
| **21** | **How confident are you in conducting a literature search and review?**   - Not at all confident - Slightly confident - Moderately confident - Quite confident - Extremely confident |
| **22** | **How confident are you in choosing the appropriate study design and research methodology for your project?**   - Not at all confident - Slightly confident - Moderately confident - Quite confident - Extremely confident |
| **23** | **How confident are you in writing a research proposal for your study?**   - Not at all confident - Slightly confident - Moderately confident - Quite confident - Extremely confident |
| **24** | **How confident are you in determining the target population and choosing the appropriate sampling technique for your study?**   - Not at all confident - Slightly confident - Moderately confident - Quite confident - Extremely confident |
| **25** | **How confident are you in choosing the appropriate data collection method for your study?**   - Not at all confident - Slightly confident - Moderately confident - Quite confident - Extremely confident |
| **26** | **How confident are you in conducting data analysis for your study?**   - Not at all confident - Slightly confident - Moderately confident - Quite confident - Extremely confident |
| **27** | **How confident are you in your ability to present and visualize the results of your study?**   - Not at all confident - Slightly confident - Moderately confident - Quite confident - Extremely confident |
| **28** | **How confident are you in interpreting the results of your study?**   - Not at all confident - Slightly confident - Moderately confident - Quite confident - Extremely confident |
| **29** | **How confident are you in writing and submitting a research manuscript for publication?**   - Not at all confident - Slightly confident - Moderately confident - Quite confident - Extremely confident |
| **30** | **How confident are you in critically appraising published research articles?**   - Not at all confident - Slightly confident - Moderately confident - Quite confident - Extremely confident |
| **31** | **How confident are you in presenting research findings at conferences?**   - Not at all confident - Slightly confident - Moderately confident - Quite confident - Extremely confident |
| **32** | **How confident are you in using softwares like SPSS, EndNote, or Mendeley Reference Manager?**   - Not at all confident - Slightly confident - Moderately confident - Quite confident - Extremely confident |
